# Book Reviews

**Published:** 1831-07

**Authors:** 


### CRITICAL ANALYSES.

Quæ laudanda forent, et quæ culpanda, vicissim

Illa, prius, cretâ; mox hæc, carbone, *notamus*.—PERSIUS.

*Cholera, its Nature, Cause, and Treatment; with original Views, Physiological, Pathological, and Therapeutical, in relation to Fever; the Action of Poisons on the System, &c. &c. To which is added, an Essay on Vital Temperature and Nervous Energy; explanatory, more particularly, of the Nature, Source, and Distribution of the latter; and of the Connexion between the Mind and the Body, &c. &c.* By Charles Searle, Surgeon, of the Hon. East India Company's Madras Establishment.—8vo. pp. 255. J. Wilson, London.

At a time when cholera is making such fearful strides, when, not content with traversing the peninsula of Hindoostan, nor satiated with ravaging Cochin-China and Tonquin, nor wearied with spreading desolation through Mesopotamia, Syria, and Persia, it has entered Moscovy, and, unaffected by the severity of a northern winter, has reared its awful standard in the heart of Russia, threatening thence to progress through Sweden, Norway, Denmark, Holland, Germany, and France, and possibly to invade our shores,—every work professing to give any account of the means by which this fell monster may be subdued, of the points in which it is most vulnerable, of the treatment by which its malignity may be lessened, must be welcomed with pleasure, and hailed with gratitude; and respect is more especially due to such as not only have the will, but also the power, to guide our at present unsettled practice to some more certain and favorable result: we must, therefore, apologise to Mr. Searle for allowing his book to remain so long unnoticed.

The essay before us is, in our opinion, one of considerable value, the mode of treatment recommended being, in several particulars, very different from that usually pursued, and the result of this treatment apparently much more successful. There are in the volume, however, various extracts, which we think might have been much curtailed, and the abridgment have been of no disadvantage: there are likewise various physiological digressions interpolated, which, fond as we are of physiology, and valuable as they may intrinsically be found, we had rather have seen in a separate publication, than mixed up with a subject of so much importance, that to it alone we would wish to give our undivided attention: these therefore we shall pass over, for the most part, *sub silentio*, and present to our readers a series of extracts from the most important parts of the volume.

Indian cholera is an instance, and not a solitary one, in which some prominent symptom has attracted too exclusive an attention, has given a name to the disease, and led to the introduction of a plan of treatment rather calculated to subdue that one symptom, as though it were the source of the evil, than to remove the actual causes, of which it should be considered only an effect.

Words, said an able physician, have killed their thousands and their tens of thousands, more than either famine or the sword; and we have often thought that the name Cholera Spasmodica, as applied to this Indian plague, has led to injudicious treatment, and countenanced, for too long time, a far too extended, we might say, perhaps, an almost indiscriminate use of antispasmodics, with opium, brandy, and other powerful excitants. For some years, however, this error has been gradually becoming corrected; and Indian practitioners, once so alarmed that they thought they could never do enough, have so far recovered from their panic, that some of the more eminent practitioners have ceased to coerce nature, and seem content to assist her curative efforts, instead of counteracting them.*

Amongst these Mr. SEARLE maintains a distinguished rank; and his work, in which, with much labour, he has collected, from various sources, authentic accounts of the several modes of treatment at different times, on different authorities pursued, substantiated with practical observations of his own, and crowned (as we may add) with personal experience

* This is well illustrated by an observation made by Mr. Searle, quoting "the words of a very intelligent friend," who says, "Our trepidation too often leads to error: changes, that can only happen after some time has elapsed, and slowly, are looked for with impatience, and if not occurring *horæ momento*, medicines to produce them are repeated in excessive doses, in short, until they aid, instead of opposing, the disease. I am sure this anxious unprofessional impatience has too much prevailed amongst us since 1817, when the cholera first alarmed us: in a word, we act from fear, and not from judgment."

of the disease, will be found a very important addition to the too meagre stock of knowledge of Indian cholera which in this country is generally possessed.

His preface announces the opportunities the author has had of becoming acquainted with the subject of which he treats, and the claims which he has upon the reader's attention, in as few words as we could well detail them in, and therefore we shall extract the passage.

"The object of this work is the improvement of our practice, in the attempt to define principles of treatment of a disease, the pathology and nature of which from being but imperfectly understood, many, very many, annually fall a sacrifice, at least such is my belief: not that I charge my professional brethren with any culpability in which I am not equally implicated; no, it was the experience of want of success in my own practice, with the loss of a relative, on the same occasion that the public sustained so heavy a one, in the death of the late revered governor of Madras, Sir Thomas Munro, (who also fell a victim to this disease,) leading me to the conclusion, that there was something radically wrong in our views and treatment, induced me to investigate the numerous public records on the subject; from which I had deduced certain inferences, and was embodying my thoughts, in a shape suitable with the intention I meditated, of submitting my views to the Medical Society of Madras, then existing, when becoming myself the subject of its attack, it not only afforded me an opportunity of verifying the conclusions I had arrived at, with regard to the line of practice which should be pursued in the treatment, but, under this personal experience, having strictly attended to the progression of the symptoms and my feelings, it gave me a clue, which enabled me to solve the difficulties I before laboured under with regard to the explanation of the symptoms and nature of the disease; in short, the explanation I arrived at, operating upon my mind with all the force of the most perfect conviction, induced me to extend my original design, and to submit my views to the press at Madras, in an essay, under the title of 'Cholera Pathologically and Practically considered.' Two years having elapsed since this period, I have had both the advantage of further experience, and time and opportunity for the maturity of my views, which, I trust, are accordingly rendered more worthy of attention; which consideration, added to some others of no less moment, induces me, to make another attempt to draw the attention of the profession to what I believe a better pathology, and to defined principles of treatment; and not to relinquish a cause, which the following quotation from one of the Asiatic Journals of last year must prove is still deserving our most serious attention: this, indeed, becomes still more apparent, when we reflect upon the cause of the disease; which, if there be any truth in the one we have assigned for it, it is but too obvious must, at certain seasons, be in operation, as long as the same laws govern nature's works:

the proofs, too, being abundant, of the disease having been known in the East, from our earliest intercourse with the country.

"At a meeting of the Medical and Physical Society of Calcutta, held on the 7th of June, amongst the papers read and discussed at the meeting, was a history of the Cholera Morbus, as it appeared recently in H. M. 14th Regiment, at Berhampore, by Dr. Mouat. Dr. M. witnessed the attacks of this scourge for upwards of ten years, and though he has studied it, watched its invasions, contemplated its progress, endeavoured to trace its causes, as well as to alleviate or mitigate its symptoms; yet he finds it the same inscrutable, inexplicable, and intractable disease, as when he first witnessed it in 1817.' This avowal, made at a public meeting, in the spirit of candour, from a gentleman, whose practice we can bear testimony to, as being in no ordinary degree judicious, but who, nevertheless, lost twenty out of ninety-four cases, requires no comment; though it renders it but too obvious, that there is abundant room for better treatment than is at present pursued: that, without any pretensions on my part to superior discernment, but having had the disease and recovered from it, which has not been the lot of many in the profession, I may be allowed to have attained a degree of experience that others who have written before me on the subject, can lay no claim to; and as I consider it an imperative duty upon every professional man, who has reason to suppose that he can throw light upon a subject which has no less to recommend it than the preservation of the lives of those committed to our charge to do so, it would be morally wrong in me, with such an impression upon my mind, to withdraw from the attempt." (*Pref.* p. v.)

The two first chapters, dedicated to the general description of the disease, and the appearances observed on dissection, being extracts chiefly from the "Report of the Madras Medical Board," Mr. Orton's work, and other authentic sources, although containing much important matter, scarcely afford any thing that requires transplanting to our pages, as all the essentially practical, and even theoretical, fruits are subsequently presented in the author's digest of the symptoms, causes, and treatment of cholera, in the seventh, eighth, and ninth chapters of his work; therefore, to the volume itself we must refer all those who wish minutely to examine the evidence and scan the facts on which it is asserted, that cholera assumes many forms, and is presented to view under many varieties, each presenting different symptoms, and raging with different degrees of severity, that in some instances purging and vomiting are the most urgent symptoms, whilst in others there is a sudden and fatal depression of the powers of life, so that the patient sinks at once, as if struck by lightning; whilst in another very numerous class of cases, cramps of the limbs, and the most terrific spasms, mark the inroads of the disease. But of these well-marked varieties, which nevertheless run most indeterminately into each other, more hereafter: we would only now observe, that these spasms, which have given a name to this disease, (Cholera Spasmodica,) and excite so much terror among the attendants, and so much solicitude in the surgeon to subdue them, mark a less fatal variety of cholera than when they are wholly absent, as our author writes.

"Mr. M'Cabe informs me, that he has found the cases which to common observation might appear the most desperate, those which were attended with spasms and retching of extreme violence, actually amongst the most tractable: a truly valuable remark, which my own experience fully confirms. Dr. Burrell saved eighty-eight out of ninety of his latter cases, and, in his general description of them, he says that the retching was constant, and the spasms so violent as to require six men to hold the patient on his cot. On the other hand, nothing can be more evident than the intractable and fatal nature of those cases in which the pulse, instead of rising, sinks at once; in which there are no spasms, and scarcely any vomiting or purging; and in which not only the secretion of bile, but all the secretions, appear to be entirely suspended." (P. 25.)

The author's own case next follows, which, however, does not seem to have been characterised by any symptoms of great severity: he probably would have recovered, if not through the means, still in spite of any of the ordinary modes of treatment. The case is given at length: we shall extract the more striking features.

C. S., "ætat. thirty-four, twelve years in India, of spare make and delicate health, felt lately tolerably well; exposed to damp air, on return home, 16th October, 1823, and likewise in his chambers, from the windows being open, from which he felt chilled.

"But in getting into bed he soon fell asleep, and continued so till about one A.M., when he awoke, suffering from a sense of distention and oppression at the præcordia, which attributing to flatulence, he endeavoured to relieve by sitting up in bed, and rubbing the abdomen; but this affording no relief, he got out of bed, with the intention of taking a little brandy and water, when he immediately felt an urgent propensity to stool, and as the commode was at hand, almost as instantly passed a copious fluid dejection, without the slightest pain or uneasiness; but with no relief to the abdominal oppression: on leaving the commode, he suddenly felt giddy, which went off on assuming the recumbent posture, but left him sighing, the pulse exceedingly weak, the voice subdued and feeble, the extremities cold and with a remarkable thrilling sensation, or feeling of nervous tremor in every fibre of the body, but more particularly experienced in the extremities: he now took half a wineglassful of brandy with double the quantity of hot water: although a little wind was expelled from the stomach, it afforded no relief to the præcordial oppression; he therefore swallowed twelve drops of oil of peppermint, in a teaspoonful of sugar; but, as no relief was experienced, warm flannels were applied to the belly; these felt hot to the part, but afforded no relief. Another copious fluid brown dejection was now passed, which was almost immediately succeeded (I believe from assuming the erect position,) by vomiting of a small quantity of mucous fluid with the peppermint: presently afterwards a desire was again felt to stool, but being restrained, the stomach appeared immediately to sympathize, and ejected with some force its contents, consisting of about six ounces of ropy mucus, enveloping a small quantity of indigested rice, which tasted very sour. The vomiting occasioned a singing noise in the ears, and sense of great exhaustion, with sighing and oppression of breathing; which was only relieved by the unceasing use of the punkah. Ten minutes afterwards the following draught was taken: ℞. Ammon. Carbon. gr. x.; Magnesiæ ʒi.; Magnes. Sulph. ʒiij.; Aquæ ℥iij. M.

"This induced an agreeable warmth in the stomach, but no expulsion of flatus, nor did it afford any relief to the præcordial oppression, which continued unabated, although hot flannels had been constantly applied. Another evacuation was now passed, and soon after a desire was again felt, but, being uncomplied with, the stomach became again sympathetically excited, and the draught, which had been taken now about a quarter of an hour, was thrown off, much diluted with a flocculent colourless fluid, tasting strong of the ammonia. Vomiting was in this instance attended with a little perspiration, but of very transitory duration; and there was a little relief felt of the præcordial oppression. A few minutes afterwards, a couple of clysters of a solution of salt in warm water were thrown up the bowels, which were succeeded by a copious flocculent mucous fluid evacuation: this afforded slight but sensible relief to the abdominal oppression. Shortly afterwards, on attempting to sit up, faintness came on, followed by vomiting of a mouthful of mucous fluid, singing noise in the ears, and feelings of great exhaustion. The præcordial oppression being still great, the clysters were repeated, and continued at short intervals, as they were generally succeeded by evacuation, which always afforded more or less relief. As the abdomen felt hot to the hand, and there was some thirst now experienced with desire for cold drink, a wineglassful of cold water was taken, and repeated every half hour with great comfort, and no subsequent vomiting. The warm salt water injections at short intervals, with the cold water for drink, and the constant use of the punkah, were continued from this time, about half-past three till six o'clock, with progressive relief to the præcordial oppression, which still continued considerable, and with general amendment, when an increase of thirst being experienced, with sense of preternatural heat of the extremities, the clysters were discontinued; and a drachm of Cheltenham salts, dissolved in four ounces of water, was taken, and the same dose repeated two hours afterwards: by which pretty copious colourless sero-mucous evacuations were continued to be passed at short intervals, with gradual relief of the præcordial oppression, and comfortable feelings of amendment. At nine A.M., a cup of tea was taken, but the milk in it soon after becoming sour, occasioned nausea and oppression of stomach, which continued a couple of hours. The heat of skin having progressively increased, at eleven o'clock the following pills were taken, with a claret-glassful of cold water; and the latter was repeated occasionally afterwards, ad libitum. ℞. Hydr. Submur., Pulv. Antim. *aa* gr. ij.; Extr. Coloc. co. gr. vj. M. fiat pil. ij.

"I must observe, that, after ten o'clock, the evacuations, which continued to be passed every half hour or so, became less copious, thicker, and somewhat of a feculent tinge: at two, they became decidedly bilious, but containing many flocculent shreds. At ten urine was voided for the first time; at about which time it was noticed by an attendant how wasted and shrivelled the fingers appeared, and on examination the toes appeared so likewise; the digits of both extremities feeling, at the same time, inflexible and contracted.

18th, six A.M. The air being calm and oppressive, the attendance of a servant to fan the patient was required throughout the night; by which he rested, and slept for a few hours at intervals, having to get up frequently to stool. The evacuations continue as limpid as water, are green, but contain some mucus and a few flocculent shreds: they are passed without griping, but occasion a good deal of irritation about the anus. Feelings comfortable, but, on rising, feels giddy and weak; pulse soft and weakly; skin a little moist. Some sago was now taken, and a dose of Cheltenham salts two hours afterwards. From this time the convalescence was rapid, the diet being light, and proportioned to the weak state of the digestive organs.

"I beg to observe that, during the whole of the time being in possession of my senses, the remedies prescribed were dictated more by my feelings than by any preconceived opinion, which soon convinced me the præcordial oppression was from the congestive state of the stomach and bowels, as the stimulants I took afforded no relief in this particular; whereas, the evacuations invariably did so, more or less: the irritating clysters were, therefore, obviously indicated not only with this intention but in relief also of the stomach, which was twice suddenly excited to vomiting, by restraining the action of the bowels: the latter is therefore, I consider, a most obvious curative indication: indeed, I felt this so much to be the case, that I pursued the hint, and have reason to suppose my rapid recovery attributable to it. Had I, on the contrary, restrained this by opium and stimulants, which has been too commonly the practice, inflammation would have become developed, with its attendant symptoms, burning heat, and extreme irritability of stomach, restlessness, and so forth; or spasms with their exhausting influence; or the absence of these symptoms, and the non-developement of excitement from cerebral congestion." (P. 41.)

"There could be no doubt regarding the nature of the attack, the diagnostic symptoms, as they have been considered, of sereo-mucous or conjee-water-like evacuations not only being present, but accompanied with the other striking characteristics of the disease, sudden invasion, extreme prostration of strength, &c. The weather, I would next observe, was wet, cloudy, and oppressive; such as usually precedes the setting-in of the monsoon, a period during which generally the cholera has been more frequently prevalent. I shall now mention as coming under my own immediate observation, and not undeserving notice, as I cannot help connecting it with the cause of my attack, a remarkable coincidence, of what may be supposed atmospheric deterioration in some way, at this time: that for a week before, there had been a singular epidemic disease among the poultry in my compound, and in the neighbourhood, not of the chicken-pox character, to which poultry are subject, but poultry, in a state of apparently the most perfect health, were suddenly seized, drooped, and died in an hour or two." (P. 42.)

"One of the dead ducks the day before was brought for my examination, when I found the whole of the inner coat of the intestines, from the gizzard downwards, in a high state of inflammation, and filled with mucus. I observed to the servants at the time, that it had died of cholera, and warned them to take care of themselves. Connecting this with the fact, not unfrequently noticed, that during the epidemic visitations of cholera, a like mortality has prevailed among cattle, atmospheric deterioration, as a cause, may be fairly inferred. Mr. Chalmers remarks, at fol. 142 of the Madras Report, "It is a curious fact, that in the towns near the hills, where the epidemic was so fatal, a disease occurred among the cattle, which kept pace with and often exceeded in mortality that of the human species." Dr. Ranken too observes, in the 74th number of the Med. and Phys. Journal, "At Rajputana, during the epidemic prevalence of the disease, the brute creation did not altogether escape the sickliness of the period; many camels, and goats in particular, having died of violent diarrhœas and other ailments.". The collector also of this district (Madura) authorizes me to assert, that in numerous instances, and from various parts of the collectorate, the like was not only reported to him, but urged by many of the riots or farmers, as a plea for the remission of their kists or revenue dues. And the same, I also heard, was the case in the Coimbatore district.

"Taking all the circumstances into consideration, I cannot help attributing my own attack, to the operation of some terrestrial exhalation, under the circumstances of exposure before mentioned; which exhalation was evidently of a local character, from its limited extent of operation; and which I cannot now help connecting with a nullah, that runs at the back of the compound in which my house was situated: ditches and the like being not unfrequent sources of malaria, which I assume to be the cause of the disease, whether I established the fact, or not, in this case; but having assumed such to be the case, I purpose, in the next chapter, to adduce the grounds on which the conclusion is founded; after making some necessary preliminary observations regarding its nature and sources." (P. 43.)

We cannot afford room to follow the author in his dissertation on Malaria, and it would be useless to quote the cases of cholera which happened at Clapham,* and presumed to have arisen from this cause, as they are already familiar to our readers. We therefore pass at once to the "Theory of the disease," in which he says that, if his views be "correct as to the cause, and operation of that cause, the symptoms which ensue are necessarily referrible to the defective excitement of the heart and brain, or principally so; these being the two principal organs, and on which the functions of the others are dependent." (P. 85.)

"Accumulation of blood takes place in the veins, which accumulation, or congestion, will necessarily be to the greatest extent at those points of the circulation the most remote from the heart's influence, which obtains, to by far the greatest extent, in the mesentric, gastric, and splenic veins, forming the roots of the vena portarum; hence the distention of the mesentric and gastric veins, and sense of præcordial oppression, the first symptom, when I was the subject of the attack, that I was sensible of." (P. 86.)

To this engorgement or congestion the sero-mucous, or conjee-water-like evacuations, are attributed, and the sickness or vomiting in the early stage referred to the stomach's defective nervous excitement.

"In a ratio with the defective excitement of the heart and brain, will be that of the glandular system: hence the diminution or suppression of the secretions of bile, urine, &c.

"Inflammation and spasm come next to be considered, and are as readily accounted for, by pursuing the same chain of reasoning, adding to the consideration, difference of temperament, idiosyncrasy, state of the individual at the time of attack, a greater or less intensity of the cause, and treatment pursued. Thus I should say, if the exudation from the mesentric and gastric vessels does not take place, or is not equal to the relief of the distended veins, free ingress of blood is not admitted from the capillary arteries, and the consequence is, they become irritated by distention and excited into inflammatory action, and hence the sense of burning heat, pain, extreme restlessness, and irritability of stomach, desire for cold water, and symptoms specifically denoting inflammation of the stomach and bowels." (P. 87.)

* Vide London Med. and Phys. Journal, for September 1828, p. 281.

"Spasms come next to be considered: they are for the most part of the clonic kind, or primarily so in all cases dependent, I believe, on congestion in the spine, at the origin of the affected nerves; and the tonic kind, to which the European is more frequently the subject, dependent on the same cause as the former, but having developed a certain degree of inflammation in the part." (P. 89.)

Cold sweat may be considered an exudation from the venous imbibing vessels, and "evinces, if not of a transitory nature, from some sudden cause of exhaustion, a still further depreciation of the heart's action, bordering, we may suppose, on its final extinction: hence it is so generally a fatal symptom, not only in this, but in most diseases." (P. 90.)

The seventh chapter contains

"Division of the disease into three species or varieties; enumeration of symptoms, division into stages, and indications pointed out to be fulfilled in the treatment of each; terminating with some important practical remarks.

"From the explanation of the symptoms of the disease, the principles may be readily deduced which should govern us in the treatment: but before entering into the subject, I beg to be permitted to divide the disease into three species, or varieties; which will bring the symptoms again under view, which perhaps is necessary, before entering upon their treatment. The first species, characterised by the severer grade of affection, I shall, with Mr. Scot, call Cholera Asphyxia. The second, presenting the common and most usual phenomena, Cholera Congestiva, as I have before taken the liberty of denominating it. The third species, in which symptoms of general excitement become developed, there will be no impropriety in classing with Cholera Morbus of nosological writers, to which it is allied, both in character and treatment.

"I have only to add, that the distinctions here made, although not without use, will not often be found so well defined in practice; as the species not only run into each other by insensible gradations, but are variously modified, by constitutional idiosyncrasy, temperament, and habits of the patient; and by numerous other circumstances of a local character; for, after all, they are but one and the same disease, modified by these circumstances and a greater or less intensity of the morbific influence.

"Cholera Asphyxia, is, as Mr. Scot observes, 'noted by the very slight commotion in the system.' The sensorium is in an instant invaded by vertigo, ringing in the ears, with deafness, and dimness of sight. The contents of the bowels, much diluted, are at once discharged, after which the white stools come on characteristic of the disease, and occasionally vomiting, but they soon terminate; a mortal coldness, extreme prostration of strength, with arrest of the circulation, come on from the beginning, and death follows, perhaps in half an hour, without a struggle; though occasionally convulsions have been noticed.

"Cholera Congestiva. The patient is, usually, suddenly seized with giddiness, borborygma, and purging; or the latter, with a sense of weakness and symptoms of indigestion, have been for some hours, or even days' duration; these are followed by vomiting, which, with the evacuations from the bowels, soon assume the conjee or barley water appearance, succeeded by great prostration of strength, tremor or twitching of the extremities, alias clonic spasm; a sunken ghastly countenance, ringing noise in the ears, cold damp skin, feeble pulse, and sense of præcordial oppression. From the sense of præcordial oppression, heat sooner or later becomes developed, and the patient complains of inward burning, attended with great thirst, and insatiable desire for cold water; the irritability of the stomach is now usually iucreased, and there is extreme restlessness. The pulse becomes now sharp, frequent, and wirey, while the extremities are cold, and in general damp: with the developement of this partial excitement, tonic spasms or cramps usually set in, commencing in the feet and legs, and gradually increasing, are extended to the upper extremities, and occasionally involves the muscles also of the belly and chest. The exhausting influence of these spasms, or sense of internal anguish, singly or conjointly, is soon succeeded by collapse; the stomach and bowels which continued before irritable, now retain whatever is poured into them; the spasms cease, the skin is livid, covered with cold sweat, and the fingers shrivelled; the eyes are suffused with blood, or covered with a dense film, half open, inanimate, and countenance death-like; coma and dyspnœa ensue, and life gradually leaves its frail tenement without a struggle.

"Cholera Morbus. The disease here so designated generally comes on with cold chills, languor, muscular pains, and sense of numbness in the extremities, giddiness or weight in the head, nausea, fulness at the præcordia, and symptoms such as in general precede an attack of fever; then follow vomiting of slimy or bilious matter, and purging of the same description, attended with griping and pain in the bowels, heat of skin, full or strong pulse; rending headach, and severe spasms, attended with excruciating pain. If this stage is not promptly subdued, it progressively or suddenly lapses into the collapse of the former variety." (P. 94.)

"Each species is made up of an assemblage of the three following stages, excepting the first, which appears to be but of one, overwhelming the system at a blow. The first stage, of Torpor or Oppression; the second, of General or Partial Excitement; and the third, of Collapse.

"The 1st variety of the disease, or Asphyxia species, would appear a simple and concentrated act of oppression, perfectly apoplectic.

"The 2d species, or Congestive; combining the whole. In the first instance oppression, bounded by the tonic spasms, and other symptoms, denoting the partial or topical excitement of the second stage; which terminating in the last of collapse, is evinced by the spontaneous cessation of the vomiting, purging, and spasms; accompanied with the loss of pulse, and profuse cold sweat.

"The 3d, or Cholera Morbus, partakes of the first stage of oppression in a slighter degree; the second of excitement being its more obvious and principal characteristic, but the supervention of the third stage is to be borne in mind, as an occasional consequence.

"The indications of treatment resulting from thus viewing the disease, are obvious; to remove the first stage of oppression, which our explanation attributes both to suppression of power and venous congestion, by remedies both stimulant and evacuating; then follows, on its supervention, the moderating or removing the second, of excitement, by remedies more particularly evacuating; bearing in mind, at the same time, the nature of the disease, that the powers of life may not be, by the means employed in this, fatally subdued, in the event of the third, of collapse, ensuing: in which stage the indications are, to allay irritation, restrain every debilitating evacuation, to restore the natural secretions, and, at the same time, to husband and support the remaining feeble powers of existence." (P. 97.)

Previous to the detailed account of the treatment, Mr. Searle very strongly impresses the propriety of keeping the patient still, and in a recumbent posture.

"This cannot be too strictly enforced, as the following will prove, two patients having under my own immediate care lost their lives, from neglect in this particular: the first I was supporting in my arms and administering to him some warm soup; syncope at the time he was drinking it ensued, and although I immediately laid him down, poured within a minute some æther in his mouth, and adopted every other expedient that would suggest itself in such a case, it was all to no purpose, the spark of life could not be recalled. The other patient was a Sepoy, who had completely got the better of the attack, and who felt so well, that he got up with the intention of relieving his bowels outside the hospital, at least so I was told, but before he reached the door he fell down faint, and, as the dresser was not at hand, he died before assistance could be afforded." (P. 99.)

"The immediate causes of the disease being (contended to be) the torpor of the general capillaries of the system, by which the chemical changes in the blood are but imperfectly effected, the primary indication of treatment is the restoration of excitement to these vessels;" and, for the fulfilment of this indication, calomel, in large and repeated doses, is prescribed, aided by stimuli, both general and local, saline enemata, &c.

"With the same intentions, added to some others, bloodletting becomes a remedy of great importance; it would appear by the removal of oppression from the capillary vessels, to afford direct aid in their excitement, or necessarily to increase the circulation through them, as verified in daily experience, the blood flowing in common phlebotomy becoming after certain loss, of a brighter colour; which can only happen, from the removal of resistance from the veins to its ingress from the arteries. It is hardly necessary to add the more obvious indications for bloodletting in the second stage of the disease." (P. 103.)

"But, in saying thus much in its favor, I must add my most solemn protest, not only against its indiscriminate employment, but in any one case, usque ad deliquium, which has been the advice of some, or to an extent bordering thereon, for it is not in subduing, but with the intent of exciting, the heart's action, that we resort to it in the early treatment of this disease: that to adopt it successfully, we should draw it from a small orifice, the patient being in a recumbent posture, at the same time with our finger on the pulse, that its effects may be carefully watched, carrying it to an extent limited alone by the constitution of the patient, on rising of the pulse under its loss, or arresting it should the pulse flag under the operation; though after an interval, on the pulse's restoration, it may be, where the symptoms of oppression still indicate its necessity, again resorted to, under the same restrictions, with the happiest effect.

"If proper measures have been pursued in the first stage, there will be little to apprehend on the developement of the second, or stage of excitement, beyond ordinary symptoms of febrile commotion, which to a certain extent, is common I believe in every case, proving its origin, in common, and connexion with the fevers of this class; but where it is otherwise, the patient not having come sufficiently early under our treatment, and symptoms of partial excitement ensue, evinced by tonic spasms, sense of inward burning, pain, great irritability of stomach, extreme thirst, desire for cold water, restlessness or delirium; these symptoms, originating in topical engorgement, clearly point out the indications to be pursued; viz. bleeding, purging, and calomel, to which we may add clysters, leeching over the seat of affection, and sinapisms or blisters as derivatives to the extremities." (P. 104.)

"The stage of collapse, if the patient has been timously seen, and the proper treatment pursued, I should say can never happen; but when it is otherwise, it is in such cases a little opium may become a useful auxiliary in our treatment; but as I protest against its use in the other stages in which, from a mistaken view of the disease, it has been almost of universal adoption, I have thought it necessary to enter somewhat largely into the operation of this remedy on the system, making it a distinct subject of inquiry in the following section." (P. 107.)

For this digression on the employment of opium, we must refer to the work.

In the Appendix, a large mass of most interesting matter has been collected, and so arranged as to shew the comparative success of the different modes of treatment, and the decided advantages resulting from exhibiting purgatives and stimulants, and thus assisting the efforts of nature, instead of counteracting her indications, and restraining the evacuations, by the large and overwhelming administration of opium. The Appendix will well repay the time bestowed on its perusal, and we cordially recommend the book, notwithstanding its occasional defects and minor inelegancies of style, to the attention of our readers.

"John Tennant, gunner, ætat. twenty-four, a stout healthy recruit, only five days in India, (25th of September, 1828,) was brought into hospital at half-past nine at night, with vomiting, purging, and severe cramps: states that he was attacked with slight purging about two o'clock in the afternoon, after taking some ginger beer; but had no vomiting or cramp until nine o'clock. Immediately on his admission, had a clear watery stool, in quantity of about half a pint. Has severe spasms, both of the upper and lower extremities; complains of severe pain in the epigastrium; countenance collasped and anxious; pulse 126, weak and fluttering; skin cold; thirst urgent; tongue dry. ℞. Spt. Ammoniæ Aromat. ʒss.; Tinct. Opii gtt. xl.; Mist. Camphoræ ℥ij. statim. Frictions with hot flannels and spirit of turpentine to the extremities. Vomited the draught immediately after it was taken, and along with it a quantity of clear fluid. A scruple of calomel was then given, and washed down with half a drachm of Spt. Ammon. Arom. and two ounces Mist. Camph., and brandy with warm water given occasionally afterwards.

"Half-past ten P.M. Has had no vomiting or stool since ten o'clock, but the cramps in his extremities continue very severe; pulse 126, extremely weak; skin cold, and covered with a clammy sweat; much oppression of breathing; great thirst; fingers shrivelled. Venæsectio e brach. ℞. Spt. Ammon. Arom. ʒss.; Mist. Camph. ℥ij. to be taken every half hour.

"Eleven o'clock. Only eight ounces of dark ropy blood were with difficulty extracted. Has passed one copious watery stool, intermixed with a quantity of mucous shreds; cramps continue very severe. Rept. Hydr. Submur. ℈i., et cont. alia.

"Midnight. No stool since eleven o'clock: cramps relieved; skin cold and clammy; pulse 108, feeble, but distinct; much anxiety and restlessness; thirst urgent; has passed no urine since his admission; complains of heat.

"Two A.M. Lies apparently asleep, with his eyes half open. No stool, but vomited a quantity of watery fluid; has slight cramps in his toes and fingers; pulse rather better volume; skin cold. Rep. Calomel ℈i.; continue brandy with warm water.

"Four A.M. Pulse 112, and increased in volume; skin warmer; countenance somewhat more animated; one stool of thin conjee appearance. Had slight cramps about three o'clock, which were relieved by friction; no vomiting since last report; thirst considerable. ℞. Ol. Ricini, Aq. Menth. pip. *aa* ℥i. statim. To have a little thin arrowroot and brandy occasionally.

"Half-past six. Has had one stool of a conjee appearance, and has passed a small quantity of urine; pulse 100, of moderate fulness; skin becoming warm; thirst not so urgent. Cont. ut antea.

"Ten A.M. Has had three stools, the two first of conjee appearance, the latter fæculent; skin warm; pulse 104, and of good volume; still complains of thirst. ℞. Calomel gr. iij.; Pulv. Ant. gr. ij. quarta quâque horâ sumend.

"One P.M. Had no stool until twelve o'clock, since which time has had three of a dark green colour, but containing some fæces, and a quantity of mucous shreds; passes his urine freely; skin cool; pulse 108. Has taken a little sago, with an ounce of brandy, a few minutes ago. Cont. pilulæ.

"Six P.M. Has had two stools since one o'clock, of the same appearance, but containing more fæces; skin natural heat; pulse 104. Cont. pilulæ, &c.

"27th. Has slept tolerably during the night; two stools, of a highly bilious nature; pulse ninety-eight; skin natural heat. ℞. Ol. Ricini ℥i. statim; arrowroot for breakfast.

"Vespere. Has had four highly bilious and fæculent evacuations; skin cool; pulse eighty-eight; no particular thirst. ℞. Calomel gr. v. horâ sumend.

"28th. Has passed a good night, and had two scanty bilious stools; skin cool; pulse eighty-six; tongue clean; scrotum inflamed and excoriated; passes his urine freely. ℞. Ol. Ricini ℥i. statim; Lotio Plumbi p. d. app.

"29th. No complaint but weakness; mouth slightly sore. Convalescent.

"Another patient was admitted into hospital a few hours after this man, with precisely the same symptoms, and was treated in the same way, with the like success." (P. 227.)

---

*Treatise on the Excision of Diseased Joints.* By JAMES SYME, F.R.S.E., Surgeon of the Edinburgh Surgical Hospital, Lecturer on Surgery, &c.—8vo. pp. 163; plates. Black, Edinburgh; Longman and Co. London.

THIS treatise is offered to the profession for the purpose of calling the attention of surgeons to an operation, which seems to the author to have been unjustly neglected. Mr. SYME's opinion upon this subject rests upon the firm basis of practical experience, and it can no longer be doubted that, in many cases, excision of the diseased joint may supersede the necessity of amputation, which has hitherto, with hardly any exception, been regarded as the only safe and efficient means in such cases which did not admit of recovery. It is not, indeed, surprising that surgeons should have been unwilling to attempt the excision of diseased joints, when the serious, and even frequently fatal, effects of wounds inflicted on sound joints, were so justly dreaded, and the unserviceable condition of limbs, in which anchylosis had occurred, afforded so little encouragement to encounter the hazard of an operation, which seemed to promise this as its most favorable result. In order to decide how far these objections are valid, Mr. Syme first shews what cases require and admit the operation of excision, and he states that "by far the greatest number is presented by those affections of the joints which are comprehended under the general denomination of *white swelling*."

In the first chapter, a brief description is given of the different forms of this disease, and of the treatment found most beneficial; but, without dwelling upon this preliminary discussion, we pass on to the more immediate subject in view, "the excision of the joints as a remedy in white swelling, and its merits in comparison with amputation." The advantages of amputation are, that it quickly, easily, and effectually removes the disease; but these are balanced by the serious objection that the patient is deprived of a limb; and besides it must be added, that the operation is not free from danger. In addition to the ordinary bad consequences of amputation, the author particularly notices the risk of inflammation and suppuration of the lungs, or other internal organs, which renders the result of amputation for caries so unsatisfactory, especially in hospitals. It is also observed, that adult patients, who have suffered amputation for caries, often fall into bad health, and die of dropsy, or some other chronic complaint, within a year or two after the operation.

"The great recommendation of excision is, that it saves the patient's limb; and the benefits accruing to him from this are so important and conspicuous, that, unless the objections which can be urged against it should appear, after mature consideration, to be very serious indeed, we ought not to hesitate in giving it the preference. These objections, so far as I have been able to ascertain, are the following: 1st, the difficulty of the operation; 2d, its danger; 3d, the useless condition of the limb in which it has been performed.

"In taking into consideration the difficulty of the operation, it must be ascertained, in the first place, what is requisite to constitute its effectual performance; in other words, how far it is necessary to take away the diseased integuments, synovial membrane, articulating cartilage, and extremities of the bones. In cases of old standing, where the sinuses are numerous and the suppuration has been profuse, the integuments surrounding the joint often retain hardly any trace of their original appearance or structure. They lose their laxity and mobility, from effusion of lymph into the subjacent cellular substance; become smooth and shining on the surface, which is often a dark-red or purple colour; and are so soft, that, if stitches are introduced to approximate the edges of an incision made in them, the threads instantly cut their way out. It might, therefore, be supposed that no healthy union or permanent cure could be obtained if parts in such a morbid state were allowed to remain, and that, consequently, the operation could very seldom be practised with propriety. Experience, however, has shewn that this is not the case: and that in a very few days after the operation, when the swelling and inflammation immediately consequent upon it begin to subside, the diseased integuments regain their natural characters, and ultimately become perfectly sound." (P. 16.)

Mr. Brodie believes that, when once the synovial membrane has been completely altered, it cannot be restored; but "it is proved, beyond dispute, by the facts hereafter to be mentioned, that the synovial membrane, though thickened and gelatinized to the utmost, affords very little obstacle to recovery, since it speedily disappears, partly by sloughing, but chiefly through the absorbent action of its own vessels, during the copious suppuration which ensues." The operation of excision essentially requires the removal of the whole cartilaginous surface; for although it might be expected that no harm could result from leaving any part of it that remained sound, it has been found that, when any portion of the articulating surface was left, the disease required a subsequent operation. Lastly, as to the bone: one not acquainted with the pathology of the osseous tissue, who examined the bones of carious joints after maceration, might be apt to suppose that the diseased part could not be removed without sacrificing so large a portion of the whole as to render it useless and unworthy of preservation.

"From not distinguishing between the truly diseased bone and that effused in consequence of its irritation, it appears that a much larger portion has been taken away in some of the cases of excision hitherto published than there was any occasion for. Less than a half of the portions of the humerus and femur which were removed by Moreau and Crampton, I should certainly think, so far as can be judged from the evidence of their drawings, would have been sufficient for the purpose, in which case it is plain the limbs would have been much less shortened and weakened, and the magnitude and consequent severity of the operation diminished. As already stated, the caries seldom goes beyond the epiphyses, which are all the part of the bone that the surgeon requires to remove, except in the rare cases where the bone is found to be more extensively affected; and in these it will probably be most prudent to perform amputation." (P. 22.)

Having analysed the operation, and shewn that all it essentially requires is the removal of the articulating epiphyses, the next question considered is, by what means is this to be accomplished? It is difficult to divide bones by a common saw, unless they are fairly exposed and held steady; and as these advantages cannot be obtained in operating on the extremities forming a joint, various modifications of the saw have been contrived: but if it were absolutely necessary in any case to use a saw for this purpose, Mr. Syme would prefer the common one.

"But fortunately we are not reduced to this disagreeable alternative, since the cutting pliers, which have been introduced into operative surgery with so much advantage by Mr. Liston, enable us to attain the object in view with perfect ease, whenever the ordinary saw is not applicable. That there is no difficulty at all in the operation of excision, it would be absurd to affirm; and that in some joints, particularly in certain states of disease, it is extremely perplexing, I am ready to admit; but when the object to be gained is the saving of a limb, the trouble or difficulty of its attainment ought not to be considered an objection; and from what has been said, it will, I trust, appear that there is nothing required in this operation except what a moderate share of dexterity and coolness is sufficient to achieve." (P. 24.)

With respect to the *danger* of the operation, Mr. Syme observes that there is no parallel between a wound inflicted on a sound joint, and that by means of which a carious articulation is cut out; for, in the former, unless the solution of continuity heals by the first intention, inflammation must necessarily supervene, when all the complicated, extensive, and irritable apparatus of articulation will be ready to suffer, with its usual intensity and constitutional disturbance.

"In the latter case, the joint is already open, there being always one or more sinuses leading into it, in the advanced stage of the disease, which renders the operation of excision warrantable, so that the wound which the surgeon makes cannot, merely on account of exposing the cavity of the joint, be the cause of inflammation; and even though inflammation were to happen, its bad effects would be comparatively inconsiderable, since the structure which is so apt to be violently affected is removed by the operation. But this is not all; for the effect of cutting out the diseased parts is rather to allay than excite irritation, both by removing a source of constant gnawing pain, and at the same time freeing the limb most effectually from tension. Hence patients have been observed to sleep better the night after the operation than for a long time previously. It also ought not to be forgotten, that the question here is, not whether the cutting out of diseased joints be attended with any danger, but whether this operation is more dangerous than amputation? and if, in addition to what has been said already, it be kept in mind that in excision the great nerves, arteries, and veins are not divided, that there is hardly any loss of blood, and that the system is not subjected to the disturbance which results from taking away a large part of the body at once, it seems to me not unreasonable to conclude, that the danger is greater in amputation. But experience here too may assist in settling the point. I have cut out fourteen elbow-joints, and the operation has been performed in Edinburgh three times by other practitioners: of all these seventeen cases only two have terminated fatally; and in one of them the patient would, I believe, have died from any operation whatever, while in the other the disease was found so extensive as to render the excision almost impracticable. I believe the result of seventeen amputations in similarly unfavorable constitutions would not be so satisfactory. I am aware that some may think it unnecessary to prove that the risk attending excision is not greater than that of amputation, since the great advantage of saving the limb might be thought sufficient to counterbalance some additional risk; but I have no hesitation in declaring, that, in such circumstances, I do not think a surgeon would be warranted in recommending the operation. The great object of medicine is the preservation of life, and the patient's mere convenience ought always to be reckoned a secondary consideration. Thus, if the fair induction from extensive experience should satisfy us that the limbs of ten persons labouring under diseased joints might be amputated with the probability of saving the lives of nine out of the whole, while excision of the joints would probably prove fatal to two, so that only eight would recover, though the condition of the eight would doubtless be preferable to that of the nine, I do not think this advantage ought to be regarded as sufficient to balance the life that would be lost." (P. 25.)

The last objection that has been alleged against excision, is that the limb thus preserved is useless, and not worth the pain or trouble required for its preservation. It has been said that, after the joint is cut out, the bones must either unite together, so as to render the limb rigid and unserviceable, or, if it remains moveable, the attachments of the muscles having been separated, it must be no less unfitted for use, by its flaccidity and want of subjection to voluntary motion.

"With regard to the first of these events, I think it cannot be denied that anchylosis of the shoulder or elbow, provided the other joints remained entire, so far from rendering the limb useless, would not prevent many of its usual actions, and certainly not to the extent of permitting it to be compared, in respect of utility, with an artificial substitute. But it has been ascertained by the sure decision of experience, that true anchylosis or osseous union does not occur generally or even frequently in these circumstances; indeed, I feel authorized to say, not without very great attention on the part both of the surgeon and patient in favoring its accomplishment, particularly in preserving absolute rest; but when no such precautions are used, the union is established by means of a tough, flexible, ligamentous-like substance that permits the bones to be used with more or less freedom, according to the exercise which they are made to undergo during the process of healing. And the voluntary motion, though at first impaired or altogether lost, owing to the relaxation of the muscles, which is caused by the approximation of their attachments, necessarily resulting from the shortening of the bones, gradually returns, and ultimately becomes as strong as ever. What seems to occasion the greatest difficulty in conceiving the possibility of recovering voluntary power over the new joint, if joint it may be called, proceeds from inattention to the fact, that muscles or tendons, when cut away from their attachments, fix themselves to the parts on which they come to rest. Thus the muscles of a stump adhere round the bone, so as to enable the patient to use it with force and freedom; and when amputation is performed through the tarsus, the tibialis anticus and extensors of the toes fix themselves so as to counteract the extensors of the heel. Independently of theory, however, we have here the more satisfactory assurance of positive facts; and the cases related below, will, I trust, be considered sufficient evidence to shew that it is possible to save limbs by excision of diseased joints, nearly, if not altogether, as useful as before they suffered from disease.

"In addition to the arguments against excision which have now been considered, it has also been objected that the operation affords no assurance against a return of the disease; but as this objection applies equally to amputation, it need not be taken into account." (P. 28.)

After having considered the objections that have been urged against the operation of excision, the author makes some general remarks on the mode of performing it, and on the treatment to be followed subsequently. As the operation is painful and tedious, the patient must be placed in that position which most completely exposes the articulation, and can be preserved steadily with least inconvenience. Mr. Syme has never found it necessary to apply a tourniquet; but if the patient is very weak, and the loss of a small quantity of blood is of consequence, there can be no objection to its application. A plate is given of the knife that is best suited for the performance of the operation.

"The preliminary incisions ought to be free, and so directed as to facilitate as much as possible the exposure and removal of the ends of the bones. In making them, the knife should be thrust at once into the joint, and afterwards carried close down to the bones, which is much better than cutting by degrees, as it shortens the operation, lessens pain, and renders the line of direction of the incision more determined. It is always necessary, of course, to divide more or less of the muscular and tendinous parts; but they ought to be as little injured as possible, and the most effectual method of saving them is to cut them close away from their attachment." (P. 32.)

Mr. S. states that it is impossible to form any idea of the kind of difficulty which is encountered in the operation, from trials made on the dead subject, where the parts are not diseased. The thickening and condensation of the cellular tissue, together with the gelatinous synovial membrane, which invests the articulating extremities of the bones, and fills the space between them, throw many obstacles in the way of an unpractised operator, by disguising the different tissues, and making them into one, so that the articulation requires, as it were, to be carved out of a homogeneous mass.

The common amputating saw is thought to be the best for removing the bones, and, instead of cutting completely through, "it is often better to divide the bone only partially with the saw, and then resort to the cutting pliers, which readily detach the fragment as soon as there is a groove formed for the reception of their blades. Unless the bone is very large and hard, the pliers are of themselves sufficient for the purpose." When all the diseased bone is taken away, if there are any large masses of gelatinous substance which can be easily detached, they should be also removed, as they might prevent the edges of the wound from coming readily together. The hemorrhage is generally pretty free in the first instance, but it seldom persists so as to require the application of ligatures; but if, after the operation, any vessels should continue to throw out a jet, they ought to be secured, as effectual pressure cannot be applied, and much inconvenience is apt to result from the cavity becoming distended with blood.

"The next part of the process is to place the edges of the wound in contact, and retain them together, which is best effected by the interrupted suture, unless the integuments should be so very soft as to give way under the pressure of the threads, in which case compresses of lint must be used in their stead. It is always of most consequence to unite the edges of the transverse incision, if there is one, since, if they do not heal by the first intention, they are afterwards brought together with very great difficulty, and the broad cicatrix which results from their separation is very adverse to the mobility of the joint. Some compresses of lint ought to be applied over the flaps, and then the limb being placed in a proper position, that, namely, in which it will most frequently be required after the cure is completed, it ought to be enveloped with a long roller, which affords the requisite support much better than splints or rigid cases of tin or pasteboard." (P. 35.)

The constitutional disturbance is generally very slight after the operation.

"In the course of a few days, the discharge, which was at first copious and offensive, begins to diminish; all the clots of blood issue from the wound; the swelling subsides; and the favorable change is altogether so sudden and satisfactory, as to surprise those who are not accustomed to witness the operation.

"During the cure, every means is to be employed either to keep the limb perfectly quiet, to favor anchylosis, or exercise it in the degree and extent of mobility which will be required of it. The wound is generally very nearly healed in the course of a few weeks, but one or more sinuses continue to discharge for months, or even a year or two. Small portions of bone also occasionally come away; but if the surgeon has done his duty in the first instance, he need not be under any apprehension on these accounts; and the patient will be too well pleased with being freed from the pain of his disease, and having regained the use of his limb, to feel annoyed by the trifling inconvenience which he thus experiences." (P. 36.)

In the fourth chapter, the author treats upon excision of the shoulder-joint, and relates two cases of this operation. Mr. Syme thus describes the condition of the first patient, four years and a half after excision of the shoulder-joint had been performed.

"Instead of being a thin, exhausted, anxious-looking creature, who seemed about to sink under her sufferings, she is now a stout, active, young-looking woman for her time of life: she manages the whole of her domestic concerns, and performs all the manual offices which the wife of a tradesman is accustomed to do: she sews, knits, and washes: she carries a full pitcher of water, a basket, or any other ordinary load, with the left arm. There is no discharge, pain, or uneasiness of any sort. The left arm is about an inch shorter than the right; a difference which is best observed when the two arms are viewed from behind, while the elbows are bent; indeed, it can hardly be remarked in any other position. When the shoulder is examined without any covering, it exhibits a very deep, irregular, unseemly cicatrix, owing to the great increase which has taken place in the subcutaneous adipose tissue since the operation.

"The joint, if it may be so called, allows the limb to be moved in all directions to nearly the natural extent, but her voluntary command over it is much more limited. She can move it across the chest, both forwards and backwards, with considerable force and freedom, but she has very little power of abduction; this, however, gives her very little inconvenience, as, when she wishes to separate the arm from the side, she easily does so with the assistance of her left hand. The result of this case far exceeded my expectations, and afforded great encouragement to persevere in the practice." (P. 57.)

The second case terminated fatally, but this result cannot be regarded as unfavorable to the operation itself, as the patient had previously laboured under severe pulmonic disease, and, upon dissection, his lungs were found to be almost entirely destroyed by suppuration.

The subject of the next chapter is excision of the elbow-joint. Fourteen cases are related: twelve of these patients recovered favorably from the operation, and gradually regained considerable power of moving and using the limb; the ninth and thirteenth terminated fatally. The subject of the former was a very unfavorable patient, and Mr. Syme believes that any operation, however slight, which would have disturbed the constitution, would have given rise to equally disastrous consequences; but this extreme tendency to disordered action could be learned only when it was too late. In the thirteenth case, the disease was very extensive, and the constitution of the patient unhealthy and feeble.

In the subsequent parts of the work, Mr. Syme treats of excision of the joints of the inferior extremities, and of partial amputation of the foot. The two cases of excision of the knee-joint which are described, are published in our Journal for May 1830.

Many doubts and apprehensions have previously existed as to the performance of the operation of excision in cases of diseased joints, but this treatise must completely satisfy the most sceptical and timid surgeon, that the practice adopted by Mr. Syme is free from the great hazard which has been presumed to attend it, and that the success resulting from it is such as to justify its frequent adoption. Mr. Syme takes a very dispassionate view of the subject: he meets the objections fairly that have been urged against the excision of diseased joints, and he proves (to our satisfaction, at least,) by argument, and, what is of much more weight, his repeated experience, that none of them are of sufficient importance to prevent the performance of the operation in many cases, in which amputation of the limb has hitherto been had recourse to as the only alternative.

*An Essay on the Influence of Temperament in modifying Dyspepsia, or Indigestion.* By Thomas Mayo, m.d., Physician in Ordinary to his Royal Highness the Duke of Sussex, &c.—8vo. pp. 144. Fellowes, London.

Dr. Mayo admits that the general description of the symptoms and progress of the disease termed "indigestion" has been given fully and correctly by previous writers, but in one respect they have not completely satisfied his wishes. The influence of different temperaments in producing variations of dyspeptic complaints, has not, he thinks, been sufficiently dwelt upon; and to supply such deficiency, is the proper object of this essay. We fully agree with him that, "for practical purposes, there can be no *one* indigestion:" we apprehend, indeed, that no practitioner of ordinary experience will be inclined to doubt the truth of this proposition.

We do not deem it necessary to follow Dr. Mayo through the preliminary description he gives of the bilious, nervous, sanguine, and phlegmatic temperaments, and we pass on, therefore, to the "general sketch of indigestion," in which it is confessed that originality of matter is not the main object. The works of Dr. Paris, Dr. Wilson Philip, and Dr. Johnson are favorably mentioned; but still in them the author finds "but little reference to temperament, or constitution, as any ground of pathological distinctions." Now, although these distinctions may not have been so formally or so clearly insisted upon as they are in Dr. Mayo's essay, still it is to be inferred, from the works of either of the above writers, and more especially from that of Dr. Johnson, that they were perfectly aware of the ever-varying nature of dyspeptic maladies, and of the influence of individual peculiarity over the general march of such ailments, and also of the necessity of modifying the treatment according to the mental or corporeal condition of the patient to be treated.

After having traced the common symptoms of indigestion, as they are considered in the best works on the subject, Dr. Mayo proceeds to apply to the collection of symptoms thus made the principle of division which it is his peculiar object to recommend.

"*Indigestion of the bilious temperament.*" In the greater part of the direct histories given us of indigestion, the disease described as such differs but little, says Dr. M., from that which, agreeably to *his* arrangement, would be termed bilious indigestion.

"The accounting for this fact is by no means difficult: for, of all the organs immediately concerned in the process of digestion, there is no single one so evidently influential over that process, according as it works healthily or morbidly, as the liver; and it is unnecessary to add, that, in persons biliously predisposed, the influence of this organ must be proportionally augmented.

"On the other hand, the inconveniences which indigestion produces to the phlegmatic and the sanguine, are far milder than those occurring to the biliously constituted; and the symptoms of the nervous form of the disorder, though intensely severe, are in their apparent position often distant from the place really affected, and thus either lose entirely their character as symptoms of indigestion, or are traced with difficulty to that source. But in bilious indigestion, every bodily symptom is either an abdominal sensation, or so closely linked with, so immediately springing out of one, that its connexion with processes of the digestive organs cannot for an instant be doubted. Few again, who have ever felt the moral and intellectual symptoms of bilious indigestion, are long in discovering by their sensations the strict alliance in which these symptoms are placed with some morbid state of the digestive organs.

"The extreme importance of these moral symptoms would of itself justify my present principle of division; for they are connected with indigestion, not simply as indigestion, but as the indigestion of the bilious temperament; and are accordingly liable to receive very inappropriate treatment, if this distinction is not kept in view, or, in other words, if they are associated with a form of the disorder, with which, in truth, they have no alliance.

"Nor is this, indeed, a groundless precaution. No mistake is more common, than that of imputing to bilious melancholia the tendencies and corresponding treatment of the nervous temperament, and thus improperly subjecting the patient to nervous medicines, antipasmodics and stimulants.

"The question, to what extent moral defects may be subjected to medical as well as moral discipline has a most immediate reference to the above distinctions. Thus, when such defects coexist with an arrested or vitiated state of the bile in any one, much more in the biliously predisposed, they claim, on their own account, the fullest and most careful application of those medical agents, which tend to restore the free passage, and the healthy state, of the secretion; otherwise, the intellectual powers want the material condition requisite to their healthy operation." (P. 54.)

Bilious indigestion, it is said, may perhaps be most usefully contrasted with the nervous. In the latter class of cases, stimulants, stomachics, and tonics are generally useful: aperients are only valuable as they are unavoidable.

"It would appear that, in removing nervous indigestion, the stomach itself, in its sympathies and antipathies, must be primarily consulted. But in the bilious temperament, both the sympathies of the stomach, and also its antipathies, must occasionally be disregarded in the treatment of indigestion. Thus, instead of the direct application of strengthening and soothing medicines, we are here obliged often to exclude them. While, in managing nervous indigestion, we avoid irritation, sometimes at the expense of allowing constipation; but withholding aperients, on the other hand, in controlling bilious indigestion, we must assume, that the immediate comfort which may be derived to the stomach from cordials and stimulants will be overbalanced by the mischief ultimately accruing to the whole system, from an over-stimulated liver. Thus dinner-pills have given a dangerous and deceitful comfort to many a bilious sensualist." (P. 60.)

In nervous indigestion, the question of local congestion is of very secondary importance. The circulation in this temperament is over-active, rather than sluggish. But in bilious indigestion we have, at every point, to defend our patient against local congestion. Here, indeed, Dr. Mayo observes, the diagnostic of Dr. Philip, namely, tenderness in the epigastric region, is extremely indicative of the practice which he recommends, when congestion is verging upon inflammatory action. "But, if we apply leeches to the epigastric region of the nervous dyspeptic, every time that *he* expresses slight, or even acute, tenderness at that point, we shall be inflicting constant mischief."

Of all the measures by which the bilious dyspeptic may obtain both immediate relief and protection against the severer symptoms of his disorder, the frequent use of mild aperients is considered by the author to be the most important.

"A very ill-founded prejudice, is entertained against the continuous use of aperients. It is assumed that this practice implies an unnatural and artificial procedure, calculated as such to end in mischief. Those who hold this doctrine forget what are the principles on which the action of the bowels is maintained, where no medicine is used. In such cases, it is the daily food which excites the peristaltic movements, and elicits the secretions of the intestines, and thus occasions their requisite action. Now aperients do precisely the same thing; and it will be difficult, by any reasoning, to make good the supposition, that small portions of aloes, of rhubarb, of ipecacuanha, or of compound extract of colocynth, have generally a more unwholesome purgative effect, than cabbages, potatoes, and turnips. Of this point I feel certain, that the state of the intestinal canal in many nervous persons, who are so far from requiring aperients, that a tendency to irritation is constantly besetting them, possesses a more morbid character than the opposed condition of the bilious temperament. In the latter case, digestion may be very well performed, provided the aperients are well selected. In the former, or nervous case, it must frequently be hurried." (P. 62.)

Dr. Mayo speaks of melancholia as an advanced stage of dyspepsia, and he believes that this is its character in a majority of cases.

"Still there are some in which the bilious temperament would appear to conduct to melancholia without the intervention of any hitherto recognized symptoms of dyspepsia. Sometimes, indeed, I have almost thought it a matter of congratulation to persons deeply tinged with the bilious temperament, when, owing to the immediate inconvenience of dyspepsia, they have been compelled to adopt precautions, and to obey rules of diet, and medicine and place. The same kind of measures as are requisite to preserve peace and comfort in the intestinal canal, under such symptoms, are equally adapted to avert cerebral disease, where there is no precursory dyspepsia.

"In either case temperance, active exercise, and often a frequent repetition of mild aperients, are expedient, where the bilious temperament is strongly marked. Temperance, indeed, the rising from table with a stomach unoppressed, and a head not heated, is of paramount importance: and if the dark mental symptoms of this temperament are impending, such precautions are doubly requisite; for it is one of the afflicting circumstances of melancholia, that it deprives its victim of the moral energy by which alone he can persevere in measures requisite for his cure. In other diseases it is hard to find the appropriate remedy; in this, it is equally hard to apply the remedy when it has been found. And thus it happens, that we often see the unhappy sufferer unnerved in courage and enfeebled in intellect, revolving in a tedious cycle through a long series of medical advisers, without resolution enough to pay to any one of them consistent obedience.

"With regard to the treatment of melancholia, viewed as an advanced stage of bilious indigestion, it must, from the outset of that treatment, be remembered, that the state of the patient has by that time become a very debilitated one.

"The bilious temperament is not essentially a feeble one, but he, in whom the mental disease has supervened upon dyspepsia, has become asthenic. If his powers of receiving food are not greatly impaired, his powers of obtaining nourishment certainly are. Food, except when taken in the smallest quantities, generally oppresses him from the moment at which he has taken it, until some rapid aperient has freed him from it; and this state has, in most cases, continued long before the mind obtains attention as a seat of disease.

"The risk of depletory measures, as tending to convert this secondary affection into an almost incurable state, the *démence* of the French writers, has accordingly become extreme. The lancet has no place here. The use of mercurials requires perseverance indeed, but caution and moderation. I have seen them, when pushed to salivation, change perversion of intellect into hopeless fatuity. This caution is the more required, in regard to our present subject, because melancholia or hypochondriasis, when a primary disease, and not the sequel or advanced stage of dyspepsia, bears on the whole more active depletion, than that acute and noisy form of insanity which belongs to the nervous temperament." (P. 70.)

In considering this subject, Dr. Mayo takes the opportunity of recalling an opinion which he formerly adopted and published on an important point connected with it.

"The error which I have to admit was that of inculcating a depletory process in certain forms of mental derangement, without recognizing an important principle, which subsequent experience has made clear to me; that in every form of this disease a greater and more depressing effect is produced on vital power by processes of depletion, than the analogy of other diseases would lead us to expect. Farther observation has convinced me, that such symptoms of apparent fulness or plethora in the patient, and of action in his pulse, as would warrant active depletion when the symptoms of insanity do not exist, would not always authorize it under the presence of that disease.

"The vehement irritation proceeding in mania, which we may denominate nervous derangement, and the state of distressful and bewildering thought, which belongs to melancholia, amply account for this want of power.

"Such seems to have been the practice of the late Dr. Gooch, although he does not assign his grounds for it. After considering puerperal insanity as an asthenic disease, he thus generalizes his remark: "I would lay down this rule for the employment of bloodletting, never to use it as a remedy for disorders of the mind, unless the disorder is accompanied by symptoms of congestion, and inflammation of the brain, such as would lead to its employment if the mind were not disordered. Even here, however, great caution is necessary; local is safer than general bloodletting." (P. 73.)

In the next chapter, indigestion of the nervous temperament is described. The observations which Dr. Mayo offers upon this part of his subject are by no means devoid of interest. The practical point particularly insisted upon is the necessity of abstaining from any active and debilitating mode of treatment in patients who are very easily excited, and whose vital powers are speedily exhausted.

In the sanguine constitution, the physician is more strongly tempted to allow himself a loose and careless practice in regard to the medical management of the case, than in any other temperament. "We are here aware that we are drawing upon a large bank, and we are proportionately lavish in our drafts. We choose to overlook the fact that every expenditure of strength, which we heedlessly inflict by an unnecessary medical measure, may advance the strong, as well as the weak, into that avenue, the termination of which is death." It should be remembered, that moderate doses of aperient medicines act powerfully on the sanguine habit.

The too-prevalent custom of trusting solely to purgative medicines for the cure of dyspepsia, as it exists in persons of the sanguine temperament, is objected to. Dr. Mayo has met in practice with many persons whose digestive powers have never recovered from the rough usage which they have undergone, when the plethoric state of the system has been relieved *solely* by purgatives.

"Here a well-timed bloodletting would have been a most economical line of proceeding; for, acting in combination with a much shorter and less severe course of medicines than would otherwise be requisite, it would have spared the digestive organs that long continued attrition and excitation which has often converted acute into chronic indigestion, instead of entirely dispelling it.

"It has more than once happened to me to be requested by patients labouring under those sensations of heat, fulness, and irritability, which certainly obtain relief from aperients, to prescribe to them a loss of blood, rather than that class of medicines. They have urged, from their personal experience, that the use of purgatives will disturb that regular action which they at present possess, and occasion constipation: while a moderate loss of blood will restore them to a state of general freedom and comfort. This measure, they have also assured me, will be attended by some relaxation of the bowels, which, if effected by aperients, would be a process of much irritation. The persons from whom I have collected these remarks have been females, of a mixed nervous and sanguine temperament, and moderate in their diet." (P. 95.)

The serous or phlegmatic temperament, which is opposed to the sanguine, is considered under two points of view. One of these states is characterised by relaxation, the other by feebleness, having in common a liability to disorders of congestion, and a freedom from feverishness: differing, however, remarkably in this point, that the relaxed constitution is capable of great endurance; while the asthenic is, as its name implies, easily exhaustible.

"It has been above observed, in speaking of the sanguine temperament, that persons thus constituted are not easily set wrong, and are easily set right. Now, if we pursue this consideration through the two forms of the serous temperament, we may perhaps affirm, that the relaxed offer a difficulty in each, instead of one, of these respects. For it is equally difficult to set persons of this temperament wrong, or when wrong to replace them in a state of health. They are not easily hurried down into fever, nor raised up into strength.

"Of the asthenic again, the contrary of the state imputed to the sanguine may be taken as characteristic. They are easily set wrong, but recover a state of health slowly and unwillingly.

"Now, these two forms of the serous or phlegmatic temperament differ remarkably in the treatment, which their incidental disorders require. Very active purgation, and at the same time very active stimulation, are generally found to suit the relaxed habit. It can bear, indeed it can profit, by profuse serous dicharges. To the feeble temperament this, or any other lowering treatment, is absolutely inappropriate.

"In bearing with advantage copious purgation under dyspepsia, the relaxed branch of the serous temperament has much affinity to the bilious, with however one remarkable distinction, namely, that the immediate union of tonics and aperients is highly suitable in the relaxed constitution; whereas the more bilious his temperament, the less readily can the dyspeptic bear the constrictive effects of tonic medicines. This distinction is remarkably applicable to the use of steel. Mercurial medicines, used temperately, both relieve and excite the relaxed habit: in either effect they are beneficial.

"All that class of applications, which promote activity of circulation on the surface of the body, are applicable to this temperament; such as friction with liniments, or the flesh-brush, and cold affusion, or the shower-bath. The feeble or asthenic are sometimes depressed by these remedies; the sanguine may be over-stimulated, the nervous may be irritated by them: but the relaxed habit is simply braced and invigorated by their use.

"That persons thus predisposed should be moderate in their diet, so as not to overload sluggish organs of digestion, is a point of obvious importance. But I have had occasion to observe, that the system of dividing the allowance of food into small meals, with brief intervals, is in this class of cases, an extremely bad one. The most wholesome of stimulants, namely hunger, is thus withdrawn, where it is most wanted, and a substitute must be found in an increased quantity of wine and cayenne pepper. Besides, I have reason to believe, that the relaxed stomach, when roused by a meal as large as it will bear, is in a much more efficient and vigorous state, than the same stomach, when inadequately supplied. Let the patient, however, in order that this rule may not be abused, keep one other constantly in his mind; namely, that he should always rise from his meal with an appetite.

"It is with persons of the relaxed temperament, that a cautious economy of liquid food under dyspepsia has been found so valuable as to have given to such abstinence the authority of fashion, in a very mischievous degree. The dry stimulating food thus supplied to the mucous membrane of the stomach, compelling it to secrete its juices freely, and to perform its contractions forcibly, may well be conceived to suit the state of atony, which I impute to it. On the other hand, this dry stimulating food is calculated to inflict the severest mischief on a stomach differently constituted; one, for instance, in which the phenomena of indigestion coexist with a nervous irritability of membrane in the intestinal canal.

"The same remark applies forcibly to the childish extension of the use of the white mustard seed. If this remedy be efficacious in any case, it must also be mischievous in those cases to which it is inapplicable, namely, in the same class of cases as would be injured by the dry system of diet." (P. 97.)

Under the title of "*apparent cases of atrophy*," the student will find many hints that he will do well to impress upon his mind.

---

### COLLECTANEA.

Floriferis ut apes in saltibus omnia libant,
Omnia nos, itidem, depascimur aurea dicta.

